# Genomic Mechanisms Governing Mineral Homeostasis and the Regulation and Maintenance of Vitamin D Metabolism

**DOI:** 10.1002/jbm4.10433

**Published:** 2020-12-05

**Authors:** J Wesley Pike, Seong Min Lee, Nancy A Benkusky, Mark B Meyer

**Affiliations:** ^1^ Department of Biochemistry University of Wisconsin‐Madison Madison WI USA

**Keywords:** CYTOCHROME P450, CRISPR/Cas9, ChIP‐seq, VITAMIN D, GENE REGULATION, 1,25(OH)_2_D_3_, *Cyp27b1*‐KO, *Cyp24a1*, FIBROBLAST GROWTH FACTOR 23, PARATHYROID HORMONE, PTH/Vit D/FGF23, CYTOKINES, TRANSCRIPTIONAL REGULATION, GENETIC ANIMAL MODELS

## Abstract

Our recent genomic studies identified a complex kidney‐specific enhancer module located within the introns of adjacent *Mettl1* (M1) and *Mettl21b* (M21) genes that mediate basal and PTH induction of *Cyp27b1*, as well as suppression by FGF23 and 1,25‐dihydroxyvitamin D_3_ [1,25(OH)_2_D_3_]. The tissue specificity for this regulatory module appears to be localized exclusively to renal proximal tubules. Gross deletion of these segments in mice has severe consequences on skeletal health, and directly affects *Cyp27b1* expression in the kidney. Deletion of both the M1 and M21 submodules together almost completely eliminates basal *Cyp27b1* expression in the kidney, creating a renal specific pseudo‐null mouse, resulting in a systemic and skeletal phenotype similar to that of the *Cyp27b1*‐KO mouse caused by high levels of both 25‐hydroxyvitamin D_3_ [25(OH)D_3_] and PTH and depletion of 1,25(OH)_2_D_3_. *Cyp24a1* levels in the double KO mouse also decrease because of compensatory downregulation of the gene by elevated PTH and reduced FGF23 that is mediated by an intergenic module located downstream of the *Cyp24a1* gene. Outside of the kidney in nonrenal target cells (NRTCs), expression of *Cyp27b1* in these mutant mice was unaffected. Dietary normalization of calcium, phosphate, PTH, and FGF23 rescues the aberrant phenotype of this mouse and normalizes the skeleton. In addition, both the high levels of 25(OH)D_3_ were reduced and the low levels of 1,25(OH)_2_D_3_ were fully eliminated in these mutant mice as a result of the rescue‐induced normalization of renal *Cyp24a1*. Thus, these hormone‐regulated enhancers for both *Cyp27b1* and *Cyp24a1* in the kidney are responsible for the circulating levels of 1,25(OH)_2_D_3_ in the blood. The retention of *Cyp27b1* and *Cyp24a1* expression in NRTCs of these endocrine 1,25(OH)_2_D_3_‐deficient mice suggests that this *Cyp27b1* pseudo‐null mouse will provide a model for the future exploration of the role of NRTC‐produced 1,25(OH)_2_D_3_ in the hormone's diverse noncalcemic actions in both health and disease. © 2020 The Authors. *JBMR Plus* published by Wiley Periodicals LLC on behalf of American Society for Bone and Mineral Research.

## Introduction

**Fig 1:**
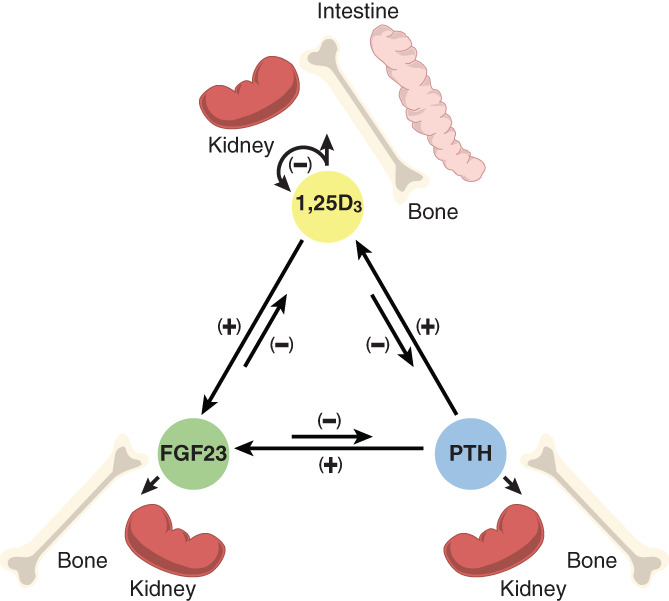
The interregulatory nature of the mineralotropic hormones PTH, FGF23, and 1,25(OH)_2_D_3_ (1,25D_3_) and their genes (via expression of *Pth*, *Fgf23*, *Cyp27b1*, and *Cyp24a1*). 1,25(OH)_2_D_3_ feedback regulates their own expression. Arrows indicate the direction and nature (+/−) of regulation. Kidney, intestine, and bone represent the direct mineral regulating targets of the individual hormones.

Biological processes integral to the maintenance of mineral homeostasis are highly complex, and likely represent one of the most exquisite regulatory systems that can be defined in higher vertebrates. The need for this regulation is quite clear: Appropriate levels of calcium (Ca) and phosphorus (P), as well as other rare nutrients, are essential for the unique functioning of many if not most life processes. Thus, aberrant levels of these elements can lead to an astounding array of human diseases. Ca and P levels, in particular, are regulated by vitamin D, PTH, and FGF23, the three primary mineralotropic hormones whose independent actions in the intestine, bone, and kidney orchestrate mineral absorption, resorption, and reabsorption, respectively.^(^ ^1^ ^)^ Interestingly, aside from their unique and frequently overlapping functions in these key tissues, as seen in Fig. 1, each hormone also coordinately regulates the production, processing, and/or activity of the other two.^(^ ^2^, ^3^, ^4^, ^5^ ^)^ An additional target is the parathyroid gland (PTG) because this organ is the sole producer of PTH. Indeed, PTH is subject to positive regulation by low Ca and negative regulation by FGF23 and 1,25‐dihydroxyvitamin D_3_ [1,25(OH)_2_D_3_] under a variety of physiological states. From a mineral homeostasis perspective, however, the dominant of the three hormones may be vitamin D, given the intricate nature of its metabolic activation, its striking regulation of both PTH and FGF23, and its broad activity profile across tissues. Thus, though each of the three hormones displays novel activities at the kidney and skeleton, and at other nonmineralizing tissues, vitamin D is alone in its capacity to induce dietary Ca and P absorption from the gut.

Vitamin D is derived through sunlight photo‐conversion from cutaneous 7‐dehydrocholesterol in the skin. Nevertheless, it must be converted via two sequential hydroxylation reactions to the active hormone, first in the liver by CYP2R1 to 25‐hydroxyvitamin D_3_ [25(OH)D_3_], though other enzymes and tissues convert smaller amounts, and then in the kidney by CYP27B1 to 1,25(OH)_2_D_3_, the active hormone, whereupon it is released as an endocrine principle into the circulation.^(^ ^6^ ^)^ Of significance, the levels of this hormone are also governed via the regulated expression of *Cyp24a1*, which initiates the eventual degradation of 1,25(OH)_2_D_3_ to calcitroic and calcioic acids via less active 1,24,25(OH)_3_D_3_ or 1,23,25(OH)_3_D_3_ intermediates.^(^ ^6^, ^7^ ^)^ Thus, 1,25(OH)_2_D_3_ levels are determined by the coordinated expression and actions of two gene products; the synthesis and degradation of PTH and FGF23 also involve actions by several different gene products. Recent studies suggest that CYP24A1 also contributes to the regulation of 25(OH)D_3_ via its actions to maintain appropriate vitamin D substrate concentrations, not through the control of synthesis, but rather through catabolism to the less active metabolites 24,25(OH)_2_D_3_ and 23,25(OH)_2_D_3_.^(^ ^8^ ^)^ Thus, the biological function of CYP24A1 may be largely to prevent an inappropriate increase in both 25(OH)D_3_ and 1,25(OH)_2_D_3_ under conditions where these vitamin D metabolites could reach toxic levels, thereby provoking potentially lethal hypercalcemia.

Much of the interregulatory nature of the three mineralotropic hormones as depicted in Fig. 1 has been defined over the past several decades. Thus, it is well known that PTH is a primary inducer of the renal expression of the *Cyp27b1* gene encoding CYP27B1 in the kidney, whereas both FGF23 and 1,25(OH)_2_D_3_ itself are strong suppressors of *Cyp27b1* expression.^(^ ^2^, ^3^, ^4^ ^)^ More recent studies have shown that the renal *Cyp24a1* gene is transcriptionally regulated reciprocally by these same hormones, driven by homeostatic responses that occur as a result of changes in *Cyp27b1* expression that link the actions of both enzymes to the maintenance of appropriate 1,25(OH)_2_D_3_ levels.^(^ ^8^ ^)^ 1,25(OH)_2_D_3_ feedback, in turn, downregulates PTH expression/secretion from the PTGs, while simultaneously inducing FGF23 expression from osteocytes in bone.^(^ ^9^, ^10^ ^)^ Like 1,25(OH)_2_D_3_, however, FGF23 feedback suppresses the production of PTH, thus providing additional transcriptional control.^(^ ^11^ ^)^ Importantly, PTH and FGF23 provide direct links to both Ca and P homeostasis, respectively, via the ability of Ca to control PTH and P to control FGF23 levels.

Despite observations over decades documenting the above complex regulatory phenomena, the genomic and molecular mechanisms that mediate these activities in vivo are only now emerging. We turned our attention several years ago toward understanding each of the molecular regulatory events that govern the expression of renal *Cyp27b1* and *Cyp24a1* and thus the production and maintenance of endocrine 1,25(OH)_2_D_3_. The goal was to define the genomic sites of action of each hormone, which we hypothesized would first reveal important initial insights and then provide an entrée into the molecular mechanisms involved. Although we began similar studies of the regulation of PTH and FGF23 genes by these mineralotropic hormones, we summarize in this article our recent efforts to define the genomic mechanisms through which *Cyp27b1* and *Cyp24a1* expression are regulated by PTH, FGF23, and 1,25(OH)_2_D_3_. We took advantage of newly established techniques that enabled an unbiased study of gene regulation entirely in the mouse. Accordingly, we first employed ChIP‐seq analysis of the kidney cortex and other tissues to identify potential sites of genomic action of key transcription factors, to characterize the epigenetic histone environment that surrounded these genomic sites and to determine chromatin/DNA sequence accessibility.^(^ ^12^, ^13^, ^14^, ^15^, ^16^ ^)^ We also extended our findings at *Cyp27b1* and *Cyp24a1* loci using several genetic mouse models wherein the overall expression of these two genes was strikingly enhanced because of highly elevated PTH and lowered FGF23 levels. Finally, we assessed the functions of these regulatory regions to alter *Cyp27b1* expression in vivo by using a CRISPR/Cas9 gene‐editing approach wherein key segments of both genes were deleted individually from the mouse genome and the regulatory, systemic, and skeletal phenotypes evaluated.^(^ ^17^, ^18^ ^)^ Utilizing these techniques, we discovered several complex distal regulatory modules that control the expression of *Cyp27b1* and *Cyp24a1* uniquely in the kidney that modulate, in turn, the blood levels of endocrine 1,25(OH)_2_D_3_.^(^ ^8^, ^19^, ^20^ ^)^

## Regulation and Maintenance of Vitamin D Metabolism

### Identifying the complex tissue‐specific regulatory module that controls renal *Cyp27b1* expression and the endocrine production of 1,25(OH)_2_D_3_

**Fig 2:**
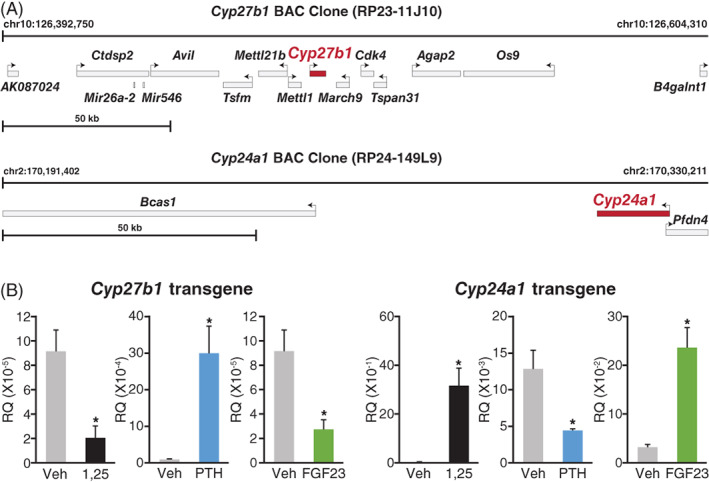
Extended bacterial artificial chromosome (BAC) clone transgenes that contain mouse *Cyp27b1* and *Cyp24a1* genetic loci recapitulate the hormonal regulation by exogenous PTH, FGF23, and 1,25(OH)_2_D_3_ regulation seen for endogenous genes in mice. (*A*) Schematic depiction of mouse transgene structures. (*B*) Hormonal regulation. Transgenic mice were prepared and selected as recently reported.^(^ ^22^ ^)^ Animals were injected with PTH (230 ng/g body weight [BW]; blue), FGF23 (50 ng/g BW; green), or 1,25(OH)_2_D_3_ (10 ng/g BW; black) and tissues harvested at 1, 3, and 6 hours, respectively. Transgene‐derived *Cyp27b1* and *Cyp24a1* transcripts were quantitated using probes that required the presence of the internal ribosome entry‐site module incorporated into the BAC clones. Data are derived from four to six mice per group, and presented as the means ± SEM, *p < 0.05.

We commenced our study of the regulation of *Cyp27b1* and *Cyp24a1* in the kidney by first establishing regulatory responses to exogenous administration of PTH, FGF23, and 1,25(OH)_2_D_3_ in vivo. These studies confirmed the reciprocal nature of the response of these two genes to PTH, FGF23, and 1,25(OH)_2_D_3_ as previously identified. We then confirmed through gain of function transgene experiments that segments controlling these features of the transcriptional regulation of *Cyp27b1* and *Cyp24a1* were indeed present and located within the extended genomic regions contained within the transgenes. Accordingly, we introduced large genetically marked BAC clone‐derived segments of DNA into the mouse genome using traditional methods (Fig. 2*A*),^(^ ^21^, ^22^ ^)^ and selected gene positive mouse strains that were then explored for their level of basal expression and regulation by PTH, FGF23, and 1,25(OH)_2_D_3_. We measured transgene‐derived RNA transcripts using novel probes that required the presence of unique sequences located within the transgenes themselves. As shown in Fig. 2*B*, examination of the output of both *Cyp27b1* and *Cyp24a1* genes confirmed appropriate and reciprocal hormonal regulation as previously observed for the same endogenous mouse genes in vivo. The expression of the *Cyp24a1* transgene was also assessed in a *Cyp24a1*‐null mouse following transgenic rescue of *Cyp24a1* expression in this *Cyp24a1*‐null mouse via a genetic cross. Transgenic expression of *Cyp24a1* in the kidneys of the rescued mouse resulted in the appearance of 24,25(OH)_2_D_3_ in the blood at levels slightly higher than those seen in normal animals.^(^ ^8^ ^)^ These levels were caused by a below‐normal expression of the *Cyp24a1* transgene in the kidneys that resulted in a paradoxical rise in 24,25(OH)_2_D_3_ as previously discussed.^(^ ^8^ ^)^ This transgenic confirmation of regulation within defined *Cyp27b1* and *Cyp24a1* loci narrowed the potential location of regulatory elements and was necessary in light of recent genomic discoveries indicating that regulatory regions for genes can occur frequently many kilobases or even megabases distal to their genetic targets.^(^ ^16^ ^)^ Our transgenic results provided the rationale for a more‐focused search for genomic elements within the surrounding loci for both *Cyp27b1* and *Cyp24a1* that could regulate the expression of both genes in the kidney.

### Identifying the sites of action of PTH, FGF23, and 1,25(OH)_2_D at the *Cyp27b1* gene in the kidney

**Fig 3:**
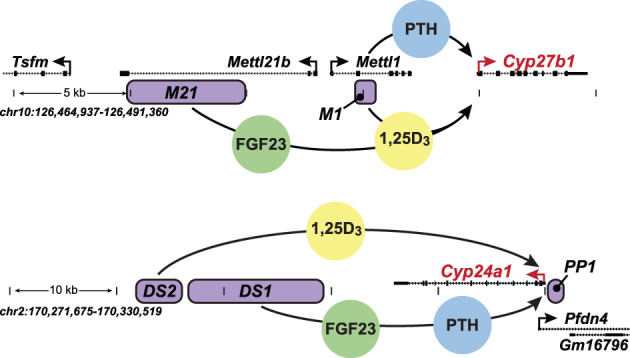
Schematic representation of the genomic enhancers for *Cyp27b1* and *Cyp24a1*. (*A*) The locations of enhancers for *Cyp27b1* are shown in purple and designated M1 and M21 with the gene dense region of the *Cyp27b1* locus, and mediated by PTH (M1; blue), FGF23 (M21; green), and 1,25(OH)_2_D_3_ (M1 and M21; yellow). (*B*) The locations of enhancers for *Cyp24a1* are shown in purple and designated PP1, DS1, and DS2 within the *Cyp24a1* gene locus, and mediated by PTH and FGF23 (DS1; blue and green) and 1,25(OH)_2_D_3_ (PP1 and DS2; yellow). Figure modified from Meyer and Pike.^(^ ^26^ ^)^

As indicated above, we utilized ChIP‐seq analysis of the kidney to identify the potential sites of action of each hormone at the *Cyp27b1* and *Cyp24a1* loci.^(^ ^19^ ^)^ An initial examination following injection of 1,25(OH)_2_D_3_ or PTH revealed the presence of four novel vitamin D receptor (VDR)‐bound sites located within the introns of the immediately upstream genes *Mettl1* and *Mettl21b* (now termed *Eef1akmt3*), sites that we designated M1 and M21 as identified in Fig. 3.^(^ ^19^ ^)^ A known mediator of PTH action via the PKA pathway, p133‐CREB (pCREB) also colocalized to these sites. Exploration of the histone environment surrounding these sites also revealed the presence of histone marks consistent with epigenetic characteristics of regulatory elements.^(^ ^23^, ^24^ ^)^ These modifications included H3K4 methylation (me1), H3K9 acetylation, and H3K36 methylation (me3). Importantly, ChIP‐seq analyses of the changes that occurred to these histone marks upon injection of PTH, 1,25(OH)_2_D_3_, and FGF23 were indicative of altered gene expression, strongly indicating that these regulatory regions were active. PTH mediated an upregulation of *Cyp27b1*, whereas FGF23 and 1,25(OH)_2_D_3_ mediated suppression. Thus, although PTH is known to activate a number of transcription factors in addition to pCREB, the presence of the VDR and pCREB suggested that 1,25(OH)_2_D_3_ and PTH were active at these four sites. In the case of FGF23, however, because the transcription factor pathways for this hormone at *Cyp27b1* and *Cyp24a1* are currently unknown, only increased epigenetic histone activity pointed to where this hormone might act. We also discovered that each of these novel sites contained an open chromatin configuration, which is essential to the functional operation of genomic control elements, an experimental result conducted in the kidney by the ENCODE (Encyclopedia of DNA Elements) Consortium via DNase hypersensitivity sequencing (DHS)‐based analysis.^(^ ^16^ ^)^ Interestingly, none of these features was present within the *Mettl1* and *Mettl21b* genes in any nonrenal tissues. This finding supports our conclusion that the regulatory module we identified is likely specific to the kidney and represents the sole determinant of unique *Cyp27b1* response to PTH, FGF23, and 1,25(OH)_2_D_3_ that links the endocrine production of 1,25(OH)_2_D_3_ to Ca and P homeostasis.

### Identifying the sites of action of PTH, FGF23, and 1,25(OH)_2_D_3_ at the *Cyp24a1* gene in the kidney

With regard to the reciprocal regulation of *Cyp24a1* by PTH, FGF23, and 1,25(OH)_2_D_3_, as depicted in Fig. 3, ChIP‐seq analysis of kidney DNA surrounding this gene's locus in mice revealed a similar overall organization.^(^ ^8^ ^)^ Thus, two extended, but separate regions downstream of the gene were observed that, in addition to well‐known promoter‐proximal sites, bound occupied clusters of either VDR or pCREB following either 1,25(OH)_2_D_3_ or PTH injection. Our earlier studies of nonrenal cells in vitro had indicated that one of these downstream regions bound the VDR and contained vitamin D response elements that were transcriptionally active at the *Cyp24a1* promoter.^(^ ^25^ ^)^ Further analysis of the kidney revealed that the chromatin state and epigenetic histone environment across these downstream regions in the kidney were also characteristic of regulatory regions and that the appropriate reciprocal regulation of epigenetic histone density was exerted by PTH and 1,25(OH)_2_D_3_ as well, indicating that these regions were transcriptionally active. Importantly, regulation of the density of these histone marks by FGF23 exclusively within the region that also bound pCREB provided unique support for a direct role for this hormone's induction of *Cyp24a1* expression, thereby providing a potential mechanistic linkage between the opposing regulatory actions of PTH and FGF23 at this gene. Interestingly, analogous to *Cyp27b1*, this downstream chromatin feature present at the PTH and FGF23 sensitive region in *Cyp24a1* locus is absent in nonrenal tissues. This provides a mechanistic explanation for why the actions of PTH and FGF23 at the *Cyp24a1* gene appear to be limited to the kidney, whereas those of 1,25(OH)_2_D_3_ itself span all tissues that express the VDR.

### Characterizing the regulatory phenotypes of mice with mutations in the *Cyp27b1* regulatory module

The identification of potential sites of action of PTH, FGF23, and 1,25(OH)_2_D_3_ at the *Cyp27b1* and *Cyp24a1* genes prompted a series of loss of function studies to characterize the specific activities of these regulatory modules at *Cyp27b1* and *Cyp24a1* in vivo and to confirm whether these modules were indeed functionally specific for the kidney.^(^ ^19^, ^20^, ^26^ ^)^ CRISPR/Cas9‐mediated gene‐editing techniques in mice provided the essential tool through which we could examine the potential functional features of these regulatory regions across multiple tissues, including the kidney. The editing technique also provided the opportunity to evaluate the phenotypic consequences that emerged following the alteration of *Cyp27b1* and *Cyp24a1* expression via these regulatory deletions, including an assessment of consequences on the production of endocrine 1,25(OH)_2_D_3_ itself. As shown in Fig. 3, we utilized pairs of RNA guides to direct Cas9 digestions in oocytes and created mice whose genomes contained an approximate 400‐bp deletion (M1) within the intron of *Mettl1* (termed M1‐IKO for M1‐intronic knockout), a 5‐kb deletion (M21) within the extended intron of *Mettl21b* (termed M21‐IKO) and a double deletion at both M1 and M21 (termed M1/M21‐DIKO). Although all three strains were indistinguishable from WT littermates at weaning, M1‐IKO and M1/M21‐DIKO mice began to exhibit retarded growth patterns early on that resulted in reduced body weight and smaller stature, which by 8 weeks reflected the physical appearance of *Cyp27b1*‐null mice. In contrast, the growth pattern and the physical appearance of the M21‐IKO mice were unremarkable relative to their WT littermate counterparts. Systemic measurements of Ca, P, PTH, and FGF23 revealed that like those of the *Cyp27b1*‐null mouse, both M1‐IKO and M1/M21‐DIKO mice exhibited hypocalcemia, hypophosphatemia, hyperparathyroidism, and very low levels of FGF23, all indicative of a potential reduction or the absence of circulating 1,25(OH)_2_D_3_. Indeed, measurements of the vitamin D hormone in the blood revealed substantially lower, but not absent levels in M1‐IKO mice and even lower levels in the M1/M21‐DIKO mice; these levels were undetectable in *Cyp27b1*‐null mice. As with the latter mice, however, both the M1 and M1/M21 deleted strains also exhibited striking changes in skeletal morphology and low BMD as previously identified. These additional systemic, hormonal, or skeletal features were absent in the M21‐IKO mice.

The underlying molecular basis for these phenotypic differences between M1‐IKO and M1/M21‐DIKO mice and M21‐IKO and WT mice emerged upon analysis of the expression patterns of *Cyp27b1* and *Cyp24a1* in the kidney. Accordingly, *Cyp27b1* expression was reduced dramatically in M1‐IKO and M1/M21‐DIKO mice; the latter strain retained a 99% reduction. A similar reduction in the level of renal *Cyp24a1* expression was also noted, a decrease that correlated directly with the absence of circulating 24,25(OH)_2_D_3_. Interestingly, although M21‐IKO mice also exhibited reductions in renal *Cyp27b1* and *Cyp24a1* expression, these decreases were not as profound. Unexpectedly, however, circulating 1,25(OH)_2_D_3_ and 24,25(OH)_2_D_3_ were either normal or slightly above normal, respectively. The striking reduction in *Cyp24a1* expression in M1‐IKO and M1/M21‐DIKO mice was also accompanied by extremely high levels of 25(OH)D_3_ relative to WT and M21‐IKO mice as well. Finally, despite the overall impact of these deletions on *Cyp27b1* and *Cyp24a1* expression in the kidney and on the phenotype of each of these mutant mouse strains, there was no effect observed on the basal expression of these two genes in nonrenal tissues such as skin, bone, intestine, spleen, or immune cells.

### Characterizing the regulatory phenotypes of mice with mutations in the *Cyp24a1* regulatory module

We also utilized the CRISPR/Cas9 approach to delete the separate regulatory regions located downstream of the *Cyp24a1* gene that bound clusters of either VDR or CREB, as illustrated in Fig. 3 and discussed above, and that appeared to mediate the expression of *Cyp24a1* in the kidney.^(^ ^8^ ^)^ Deletion of the DS1 region that mediated both the downregulation of *Cyp24a1* by PTH and its upregulation by FGF23 resulted in a significant reduction in basal *Cyp24a1* expression and complete loss of response to both hormones in the kidney, but no reduction in renal response to 1,25(OH)_2_D_3_. 1,25(OH)_2_D_3_ activity was also retained in nonrenal tissues where, as expected, PTH or FGF23 were inactive. Interestingly, deletion of the DS2 region that mediated the downstream actions of 1,25(OH)_2_D_3_ on *Cyp24a1* expression had no effect on the gene's basal expression or on its suppression by PTH in the kidney or its induction by either FGF23 or 1,25(OH)_2_D_3_. Surprisingly, however, deletion of this region decreased the efficacy of response to 1,25(OH)_2_D_3_ in nonrenal tissues such as intestine and bone. No striking phenotypic alterations were identified in either DS1‐KO or DS2‐KO mice with the exception that the loss of basal expression of *Cyp24a1* in the DS1 strain resulted in modest homeostatic compensatory changes in PTH and FGF23 levels and a reduction in the renal expression of *Cyp27b1*. This feature reinforces the idea of the reciprocal coregulation of *Cyp27b1* and *Cyp24a1* in the kidney that serves to maintain levels of circulating 1,25(OH)_2_D_3_. Therefore, our results support the observation at chromatin‐, epigenetic‐, and now gene‐regulatory levels that the downstream PTH/FGF23 regulatory module is active only in the kidney, thereby defining an underlying mechanism through which homeostatic control of *Cyp24a1* is restricted to this organ. However, it also illuminates a novel finding that the vitamin D‐regulated module downstream of the gene is also dispensable in the kidney, providing a focus on promoter‐proximal vitamin D regulatory elements, but enhances the sensitivity of induction of *Cyp24a1* by 1,25(OH)_2_D_3_ in nonrenal tissues. Thus, the coordinated expression of both *Cyp27b1* and *Cyp24a1* to maintain circulating 25(OH)D_3_ and 1,25(OH)_2_D_3_ levels in vivo is determined by common structural features within chromatin that facilitate the differential expression and regulation of *Cyp27b1* and *Cyp24a1* in either the kidney or in nonrenal tissues.

### *Cyp27b1* and *Cyp24a1* genes are regulated and functional in renal proximal tubules

**Fig 4:**
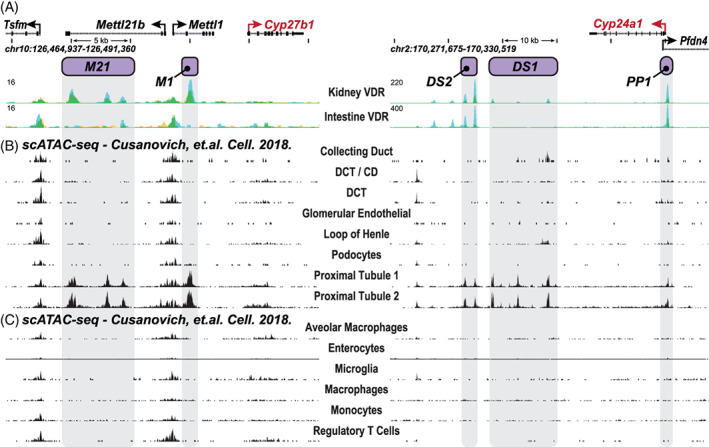
Single cell genome analyses of chromatin accessibility in renal and nonrenal cell types in the mouse. (*A*) ChIP‐seq analysis of VDR binding at the kidney cortex and intestine documenting the locations of the regulatory submodules (gray‐shaded dropdown bars) that mediate regulation by PTH (M1; purple) and FGF23 (M21, purple) as well as 1,25(OH)_2_D_3_ (M1 and M21). Data from Meyer et al.^(^ ^8^, ^19^, ^20^ ^)^ The upper panel indicates the chromosomal location of *Cyp27b1* and *Cyp24a1*, genome scale, and the identity of adjacent genes. Arrows indicate the direction of gene transcription. As seen, the overall module is restricted to the kidney, where it is dispersed within single introns of the *Mettl1* and *Mettl21b* genes, and absent in intestine (and all other tissues; see 8, 19, 20 ^)^). (*B,C*) Single‐cell ATAC‐seq (scATAC‐seq) analysis by Cusanovich and colleagues.^(^ ^32^ ^)^ Raw data were reanalyzed and displayed for only the *Cyp27b1* and *Cyp24a1* gene loci for (*B*) ATAC‐seq analysis of individual renal cell types and (*C*) analysis of representative nonrenal cell types (emphasis on immune cell types). As seen, peaks representative of an open chromatin state align with each of the individual components that comprise the kidney‐specific module (M1 and M21) and are restricted to renal proximal tubules (two separate analyses). No other renal or nonrenal cells types retain this chromatin regulatory pattern.

Although early studies suggested that *Cyp27b1* and *Cyp24a1* were expressed selectively in the proximal tubules of the kidney, respectively, more recent studies using immunocytochemical analyses have indicated that these genes, and especially *Cyp27b1*, could be produced in additional renal cell types, as well as other nonrenal cell types.^(^ ^27^, ^28^, ^29^, ^30^, ^31^ ^)^ This uncertainty drove our initial decision to explore the entire kidney as above, yet represented a potential complexity relevant to the interpretation of our initial genomic studies of *Cyp27b1* expression. This issue also raised additional biological questions relative to the linkage between *Cyp27b1* and *Cyp24a1*. Fortunately, however, recent genomic studies have been conducted by Cusanovich and colleagues on individual kidney cell isolates, as well as numerous nonrenal cell types from C57BL/6 mice using ATAC‐seq analysis.^(^ ^32^ ^)^ This approach, like DHS, reveals the presence of open chromatin sites across genomes.^(^ ^33^, ^34^, ^35^ ^)^ Several genomic data tracks from these analyses at the *Cyp27b1* and *Cyp24a1* gene loci are documented in Fig. 4. As can be seen, these analyses reveal that the regulatory sites with open chromatin features that we defined within the introns of *Mettl1* and *Mettl21b* in total kidney tissues are also evident exclusively in cells of proximal, but not distal tubule origin, or indeed in other cells of either renal or nonrenal origin. These results confirm that proximal tubules are the principle sites of *Cyp27b1* expression and the dominant source for the regulated production of endocrine 1,25(OH)_2_D_3_. Indeed, they confirm that the original identification of proximal tubules as the sources of *Cyp27b1* expression and the production of 1,25(OH)_2_D_3_ was likely correct. These data also indicate that the regulatory module we identified previously is not just kidney‐specific, but rather proximal renal tubule‐specific. Whether *Cyp27b1* expression occurs that is insensitive to PTH and FGF23 regulation in additional kidney cell types, consistent with immunocytochemical identification, remains to be resolved. Interestingly, the open chromatin states as seen in Fig. 4 that define the regulatory expression of *Cyp24a1* in the kidney are also present exclusively in proximal, but not distal tubules or the other renal cell types, as previously suggested. In conclusion, *Cyp27b1* and *Cyp24a1* are both expressed in the same key endocrine cell type in kidney, and thus capable of coordinating in real time the coregulation of endocrine 1,25(OH)_2_D_3_ production that is subsequently secreted into the blood. This has profound implications for the importance of both genes as determinants of vitamin D activation and maintenance, illustrating the power of genomic approaches for illuminating key physiological principles.

### Creation of a kidney‐specific *Cyp27b1* pseudo‐null mouse deficient in endocrine 1,25(OH)_2_D_3_

Research over the past several decades has suggested that the conversion of 25(OH)D_3_ to 1,25(OH)_2_D_3_ occurs not only in the kidney, as above, but also in a myriad of nonrenal tissues/cells (NRTCs) that include the skin, parathyroid glands, bone cells, both cardiovascular and immune cells, and many others.^(^ ^30^, ^36^, ^37^ ^)^ This idea stems from early immunocytochemical observations suggesting that CYP27B1 expression is also present in NRTCs, albeit at very low levels relative to the primary renal source. It also became evident that the regulation of *Cyp27b1* expression in NRTCs is different from that in the kidney. Accordingly, though renal *Cyp27b1* expression is tightly modulated by PTH, 1,25(OH)_2_D_3_, and FGF23, and now known to occur through the proximal tubule‐specific renal regulatory module described earlier, these mineralotropic hormones are generally inactive at *Cyp27b1* in NRTCs, where inflammatory mediators such as IL‐1β, TNFα, LPS, and certainly others function to induce this gene.^(^ ^38^ ^)^ Some insight has emerged with regard to the pathways that are involved in *Cyp27b1* induction by these inflammatory modulators, although the sites of action of these regulators at *Cyp27b1* itself have not been identified in vivo. Moreover, although it is now clear why *Cyp27b1* is regulated in the kidney, but not in NRTCs by the three mineralotropic hormones, the molecular basis for the differential basal expression of *Cyp27b1* expression in the kidney and NRTCs, although under investigation, remains unknown. Elucidation of this feature is important because it is likely integral to the evolutionary development and maintenance of the vitamin D endocrine system.

### Advancing the features of *Cyp27b1* expression and activity in NRTCs

Aside from the concept of local production of 1,25(OH)_2_D_3_, it is noteworthy that although the synthesis of 1,25(OH)_2_D_3_ in NRTCs has gained wide acceptance, fundamental insights supporting the mechanism, relevance, and biological consequence of this cellular source of 1,25(OH)_2_D_3_ production remain outstanding. Importantly, although attempts have been made to selectively delete *Cyp27b1* from specific cell types such as chondrocytes, and to assess the phenotypic consequences of these gene deletions on specific tissues, this approach has met with only modest success, perhaps because fundamental questions regarding NRTC production of 1,25(OH)_2_D_3_ in the absence of renal production remain at issue.^(^ ^39^ ^)^ Key issues pertinent to the nature of local production are as follows: (i) Does 1,25(OH)_2_D_3_ production in NRTCs occur in healthy as well as in diseased subjects in vivo, and does this local synthesis exert a measurable impact on the mechanisms of vitamin D receptor activation that both selectively alter gene expression and uniquely modify the functions of the individual cell types involved; (ii) is locally produced 1,25(OH)_2_D_3_ routinely secreted into the blood in health as has been amply demonstrated in certain human disease states; (iii) is the overall activity of locally produced 1,25(OH)_2_D_3_ influenced by or dependent upon the circulating levels of endocrine‐derived hormone; and (iv) does vitamin D supplementation and/or circulating concentrations of substrate 25(OH)D_3_ differentially impact the local versus kidney production of 1,25(OH)_2_D_3_. Resolution of these and other issues may lie at the heart of a successful vitamin D supplementation regimen capable of achieving effective therapeutic efficacy in the prevention or treatment of disease. It is noteworthy, however, that precedent has been firmly established in humans for both the production and secretion of 1,25(OH)_2_D_3_ from macrophages derived from patients with a diverse set of granulomatous diseases.^(^ ^40^, ^41^ ^)^ The elevated levels of 1,25(OH)_2_D_3_ are indeed active in these patients and exaggerate the disease by accelerating the uptake of calcium from the gut that results in hypercalcemia. Nevertheless, the overall relationship between this specific NRTC activity to produce 1,25(OH)_2_D_3_ relative to that derived from the kidney remains to be fully understood. Indeed, not all patients with inflammatory diseases present with elevated blood levels of 1,25(OH)_2_D_3_ and hypercalcemia.

### Utility of animal models to explore NRTC expression and regulation of *Cyp27b1*

It is clear that unique animal models selectively deficient in the endocrine production of 1,25(OH)_2_D_3_ will be essential for advancing our understanding of whether and how the local production of 1,25(OH)_2_D_3_ is achieved and how it contributes to biology. These models will have to be amenable to exploration into the specific issues outlined above and, in particular, to the genetic imposition of inflammatory disease states such as CKD, IBD, atherosclerosis, or perhaps even infectious diseases such as COVID‐19. Models such as the latter will enable an evaluation of the hypothesis that 1,25(OH)_2_D_3_ production is accelerated through an inflammation‐induced upregulation of *Cyp27b1* expression in the immune system, but not in kidney, for example, that will reduce the overall state of inflammation. Unfortunately, global *Cyp27b1*‐null mice are inappropriate, and efforts to create a CRE‐generated kidney‐selective *Cyp27b1*‐null mouse have thus far been unsuccessful largely because of the complexity of distinct cell types that comprise even renal proximal tubules. Regardless of whether this is achieved, however, the linkage between renal *Cyp27b1* and *Cyp24a1* expression suggests that any downregulation of *Cyp27b1* will be accompanied by a similar suppression of *Cyp24a1* expression, thereby preserving even small amounts of 1,25(OH)_2_D_3_ that might be secreted into the blood, although not sufficiently active.^(^ ^8^, ^19^, ^20^ ^)^ Indeed, it is clear that *Cyp27b1* expression is unlikely to be genetically modified and/or suppressed without the homeostatic downregulation of *Cyp24a1* by PTH and FGF23.

### The *Cyp27b1* pseudo‐null M1/M21‐DIKO mouse as a model for studying NRTC production of 1,25(OH)_2_D_3_

Interestingly, the M1/M21‐DIKO mouse described above exhibits just such a *Cyp27b1* pseudo‐null phenotype wherein the production of 1,25(OH)_2_D_3_ is strongly downregulated and incapable of maintaining normal PTH and FGF23 balance and mineral homeostasis.^(^ ^20^ ^)^ This state profoundly disrupts skeletal development and integrity, and mimics the overall *Cyp27b1*‐null mouse phenotype. Given the suppression and loss of regulation of *Cyp27b1* expression, it was surprising that even low detectable levels of 1,25(OH)_2_D_3_ were evident in the blood. Our interpretation of this finding is that though well below normal, the circulating levels of the kidney‐derived hormone still remain “inappropriately high” because of the striking suppression of renal *Cyp24a1* expression and 24,25(OH)_2_D_3_ production that is evident as a result in high PTH and low FGF23 levels. This linkage to *Cyp24a1* expression as suggested earlier, however, resulted in the absence of both 1,25(OH)_2_D_3_ and 25(OH)D_3_ catabolism in the kidney, thus raising both of these vitamin D metabolites to higher than expected levels in the blood. Given the inability of the mutated *Cyp27b1* gene in these mice to respond to PTH, FGF23, or 1,25(OH)_2_D_3_ regulation, we hypothesized that renal *Cyp24a1* levels under dietary conditions of high Ca and P exposure should be upregulated and restored as a result of the normalization of PTH and FGF23 levels, analogous to that seen in *Cyp27b1*‐null mice. Indeed, the application of this “rescue” diet to these M1/M21‐DIKO mice fully normalized the aberrant levels of high PTH and low FGF23, appropriately raised *Cyp24a1* expression without an effect on *Cyp27b1*, dramatically reduced 25(OH)D_3_ levels, and fully eliminated 1,25(OH)_2_D_3_ in the blood, as measured in the latter two cases by liquid chromatography–tandem mass spectrometry analysis. This diet also restored all the systemic parameters of normal mineral metabolism, induced genes essential for Ca and P uptake in the intestine, and rescued the skeletal phenotype as well. *Cyp27b1* and *Cyp24a1* expression in all NRTCs was unperturbed, as were genes that might be expressed as a result of the local production of 1,25(OH)_2_D_3_. These observations did not support the alternative explanation that the modest amounts of circulating 1,25(OH)_2_D_3_ in the M1/M21‐DIKO mouse were caused by the induced secretion of 1,25(OH)_2_D_3_ from NRTCs. We conclude that this rescued mouse strain, amenable to additional dietary and genetic as well as disease‐inducing manipulations, will likely prove useful in exploring key details of the NRTC production of 1,25(OH)_2_D_3_.

## Summary and Conclusions

Here we have summarized our recent work utilizing a series of genomic approaches coupled with loss‐of‐function studies, which has identified novel distal regulatory modules that mediate the reciprocal expression of *Cyp27b1* and *Cyp24a1* in the kidneys of mice. This dual regulation is essential for the control of endocrine 1,25(OH)_2_D_3_ in the circulation. Additional studies suggest that the regulated expression of both genes by these modules occurs exclusively in proximal tubules. The renal module for *Cyp27b1* is dispersed and located within specific introns in the adjacent *Mettl1* and *Mettl21b* genes in the kidney and is absent in all NRTCs. The submodule in *Mettl1* controls the upregulation of *Cyp27b1* by PTH, whereas three separate submodules in the *Mettl21b* gene control suppression by FGF23. All four submodules mediate downregulation by 1,25(OH)_2_D_3_. Thus, 1,25(OH)_2_D_3_ functions to reinforce the suppressive regulatory actions of FGF23, while opposing the inducing actions of PTH at the *Cyp27b1* gene. Of course, 1,25(OH)_2_D_3_ also induces *Cyp24a1* as well as *Fgf23*.

The renal module for *Cyp24a1* regulation is located intergenically downstream of the gene and is comprised of one segment that mediates opposing regulation by PTH and FGF23. Although this module is absent in NRTCs, a second module, which controls positive regulation by 1,25(OH)_2_D_3_, is present and active in all cell types that are targets of 1,25(OH)_2_D_3_ activity except the kidney. Deletion of the PTH sensitive component (M1) in the *Cyp27b1* gene or both components simultaneously (M1/M21‐DIKO) lead to a decrease in basal expression of *Cyp27b1* of up to 99%, and strongly reduces circulating levels of endocrine 1,25(OH)_2_D_3_. Lowered basal levels of 1,25(OH)_2_D_3_ in M1‐IKO and M1/M21‐DIKO mice lead, in turn, to reduced intestinal absorption of Ca and P, which causes hypocalcemia and hypophosphatemia that promotes a rise in PTH and a reduction in FGF23 levels, and a broad *Cyp27b1* null‐like skeletal phenotype. *Cyp24a1*, on the other hand, is downregulated by these high PTH and low FGF23 levels, which leads to high levels of 25(OH)D_3_ and a loss of 24,25(OH)_2_D_3_. Both features are responsible for the low, residual levels of 1,25(OH)_2_D_3_ in the M1‐IKO and M1/M21‐DIKO mice.

Rescue of these mice with high Ca and P diets, particularly the M1/M21‐DIKO mice, raises systemic Ca, P, and FGF23; suppresses PTH; and normalizes the expression of *Cyp24a1*. This homeostatic increase reduces 25(OH)D_3_ levels and eliminates circulating 1,25(OH)_2_D_3_. These observations provide supportive evidence for our conclusion that *Cyp27b1* and *Cyp24a1* are coregulated in the kidney, and that the residual source of 1,25(OH)_2_D_3_ in the blood of M1/M21‐DIKO mice on a normal mineral diet is not derived from NRTC sources, but rather from the kidney. Diet‐rescued M1/M21‐DIKO mice, devoid of *Cyp27b1* expression and circulating endocrine 1,25(OH)_2_D_3_, but with intact expression and regulation of *Cyp27b1* in NRTC, represent an appropriate model with which to explore features of *Cyp27b1* expression in NRTC. These include its mechanisms, substrate dependencies, role in NRTC production of 1,25(OH)_2_D_3_, and impact on noncalcemic actions in multiple tissues in both healthy subjects and in disease. Rescued M1/M21 mutant mice will be useful for exploring supplementation, the impact of 25(OH)D_3_ on *Cyp27b1* expression in renal and nonrenal tissues, and the influence of endocrine 1,25(OH)_2_D_3_ on local 1,25(OH)_2_D_3_ activation of gene expression.

**Fig 5:**
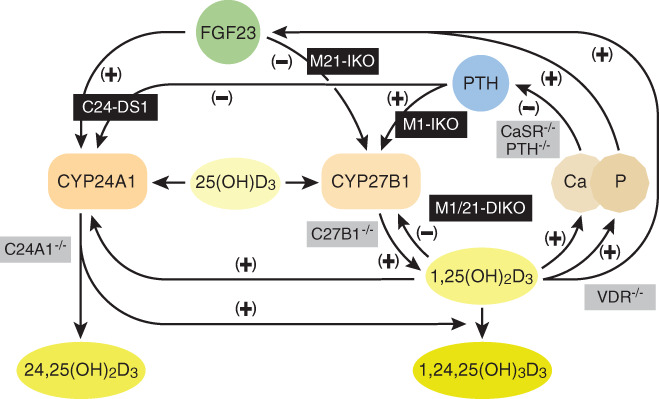
Vitamin D metabolism in the kidney. Schematic diagram depicting the regulation of vitamin D metabolism and serum calcium and phosphate homeostasis in the kidney. Our genetic models (black) and previously existing models (gray) are overlaid on or near the pathways they disrupt. Figure modified from Meyer and Pike.^(^ ^26^ ^)^

A collection of previous gene deletion models has enhanced our understanding of both vitamin D metabolism and mineral homeostasis. These individual models have been placed strategically in the context of the actions of the three mineralotropic hormones or their mediators in Fig. 5. We have now added at the appropriate interaction nodes, our newly defined mouse regulatory deletion models, providing an additional level of mechanistic complexity to the interactions that occur between the three hormones to regulate vitamin D metabolism and ultimately mineral homeostasis. Our current studies are now focused on the molecular details of the renal regulation of *Cyp27b1* and *Cyp24a1* and the genomic mechanisms through which the nonrenal expression of *Cyp27b1* activity is achieved. They are also aimed at exploiting the *Cyp27b1* pseudo‐null mouse to study the nonrenal regulation of *Cyp27b1* expression and to determine the underlying genomic mechanisms through which this is achieved.

## Disclosures

The authors declare no conflicts of interest.

## AUTHOR CONTRIBUTIONS

**J. Pike:** Conceptualization; funding acquisition; project administration; supervision; writing‐original draft; writing‐review and editing. **Seong min Lee:** Data curation; investigation; methodology. **Nancy Benkusky:** Data curation; investigation; methodology. **Mark Meyer:** Conceptualization; data curation; formal analysis; investigation; methodology; project administration; validation; writing‐original draft; writing‐review and editing.

## Authors' roles

Study design: JWP and MBM. Data collection: MBM, SML, and NAB. Data analysis: MBM, SML, and NAB. Manuscript drafting and revisions: JWP and MBM. Approving final manuscript: JWP, SML, NAB, and MBM.

### PEER REVIEW

The peer review history for this article is available at https://publons.com/publon/10.1002/jbm4.10433.
